# A deep learning model of dorsal and ventral visual streams for DVSD

**DOI:** 10.1038/s41598-024-78304-7

**Published:** 2024-11-10

**Authors:** Masoumeh Zareh, Elaheh Toulabinejad, Mohammad Hossein Manshaei, Sayed Jalal Zahabi

**Affiliations:** 1 Department of Electrical and Computer Engineering, Isfahan University of Technology, Isfahan, 84156-83111 Iran; 2 Department of Computing Science, University of Alberta, Edmonton, AB T6G 2E8 Canada

**Keywords:** Visual system, Dorsal stream, Ventral stream, Convolutional neural networks, ASD spectrum disorder, Computational neuroscience, Cognitive neuroscience, Computational neuroscience, Diseases of the nervous system

## Abstract

Artificial intelligence (AI) methods attempt to simulate the behavior and the neural activity of the brain. In particular, Convolutional Neural Networks (CNNs) offer state-of-the-art models of the ventral visual stream. Furthermore, no proposed model estimates the distance between objects as a function of the dorsal stream. In this paper, we present a quantitatively accurate model for the visual system. Specifically, we propose a VeDo-Net model that comprises both ventral and dorsal branches. As in the ventral visual stream, our model recognizes objects. The model also locates and estimates the distance between objects as a spatial relationship task performed by the dorsal stream. One application of the proposed model is in the simulation of visual impairments. In this study, however, we show how the proposed model can simulate the occurrence of dorsal stream impairments such as Autism Spectrum Disorder (ASD) and cerebral visual impairment (CVI). In the end, we explore the impacts of learning on the recovery of the synaptic disruptions of the dorsal visual stream. Results indicated a direct relationship between the positive and negative changes in the weights of the dorsal stream’s last layers and the output of the dorsal stream under an allocentric situation. Our results also demonstrate that visual–spatial perception impairments in ASD may be caused by a disturbance in the last layers of the dorsal stream.

## Introduction

The brain plays an important role in information processing by humans. It is the center of intelligence, memory, and interpretation of senses. It is also responsible for controlling and coordinating actions and movements. Studying how people and animals acquire spatial information as well as how they organize it to handle a variety of daily tasks, from recognizing a scene or a place to directing a movement into the environment, is one of the goals of the field of spatial cognition. Spatial cognition relies mainly on two frameworks: egocentric and allocentric frames of reference. The egocentric frames of reference specify spatial information depending on the observer’s position and point of view. The allocentric frames of reference encode spatial information independently of the observer’s position^1^.

The primary visual cortex processes visual stimuli in two parallel streams and generates visual perception; the ventral and dorsal streams. The ventral visual stream underlies object recognition abilities^2,3^. The dorsal visual stream processes the spatial relationships between objects and motion^4,5^. How to assess an object’s size, form, location, and motion are all covered in detail by visual–spatial perception (VSP)^6–9^. Difficulties in recognizing objects, faces, shapes, and colors are typical symptoms of ventral stream damage. Damage to the ventral stream also impairs language perception, facial expressions, reading, and visual memory.

On the other hand, poor ability to handle complex visual scenes, reduced visual field, impaired visual guidance, perception of movement, and simultaneous perception are deficits in the dorsal stream damage^10,11^. Moreover, distance perception and size constancy deficits are also related to dorsal stream damage^12^. The aforementioned visual impairments are often categorized as Dorsal Visual Stream Dysfunction (DVSD), which is an umbrella term for the group of cerebral visual impairments (CVI) degrading the way vision is created in the posterior parietal lobes^13^. DVSD is seen in several conditions such as premature birth, cerebral palsy (CP), hydrocephalus, Autism Spectrum Disorder (ASD), and Alzheimer’s disease (AD)^10,14,15^.

There have been substantial attempts in recent years to understand the structure and function of the brain due to the significance of understanding its functionality in order to analyze neuro-diseases and comprehend the connectivity of the brain areas^16–18^. These computational and mathematical models have the remarkable ability to examine the functional relevance of brain areas for human behavior, reveal how the functionality of areas changes throughout development and aging, and expose the disruption of areas in a variety of mental diseases. They also provide a good platform for validating existing intuitions about the brain. Moreover, they can help test new hypotheses and ideas about the brain and measure the results of experiments with the help of mathematical analysis^19,20^.

Some of the brain models employ artificial intelligence tools such as Neural Networks (NNs)^21–24^. Neural networks aim to solve problems analogous to the human brain. The architecture of these networks is similar to the brain. These networks consist of layers containing a population of neurons trying to perform specific tasks. The neurons in these networks, like biological neurons, learn to produce the proper output for a given input^19^. From another perspective, Multi-tasking models similar to the brain functionality try to solve problems by sharing features among related tasks^25^. Multi-task learning (MTL) is a method of training a single deep neural network to perform multiple tasks simultaneously^26,27^. Our goal is to provide a framework that advances our understanding of brain functions from both biological and computational perspectives rather than solely focusing on advancements in computer vision. We use this idea to model the operation of two visual system pathways for object recognition, categorization, and distance calculation. In computer vision, one of the metrics to calculate object distance is to use a bounding box and determine the distance to the object using the triangulation distance measurement^28,29^. Unlike computer vision methods, we aim to model the dorsal pathway with a distance estimation task. Therefore, we employed the neural layer to carry out the task of distance estimation in the model, which involves determining the distance in two dimensions by considering the color, texture, and form of objects.

Convolutional Neural Networks (CNNs) currently provide one of the most promising models for the ventral visual stream. Specifically, CNN-based models have achieved good performance in the object recognition tasks^19^. However, to the best of our knowledge, no CNN-based model has been presented to date, for the combination of the dorsal and ventral streams (i.e., in other words, a model that can not only recognize objects but can also estimate the distance between them). Specifically, our model aims to mimic two crucial functions of the human brain: 1) estimating the distance to an object, and 2) quantitatively assessing how far an object is. These functions are essential to various daily motor tasks, such as grasping and reaching^5^. Our approach emphasizes the biological and functional aspects of these tasks rather than solely focusing on numerical accuracy and performance metrics. On the other hand, the artificial intelligence models presented in the field of ASD focus on the diagnosis of ASD and do not model ASD and ASD-related disorders including the potential DVSD^30–33^. Our most prominent motivation in this paper is to find solutions for the above two problems.

In this study our main contributions are: (1) We present a visual system model that includes both ventral and dorsal streams. Thus, we achieve a more biologically plausible model of the visual system. (2) Our proposed model can mimic distance estimation and object localization as two of the dorsal stream functions. (3) Based on the proposed model, we simulate some visual impairments and investigate whether and how training may improve the symptoms of the impairments.

The rest of the article is organized as follows. In “Related works” Section, we review some related works. In “Methods” Section, we first introduce the dataset we use for training the proposed models. Then, we present a visual system model. In the continuation of this section, we simulate a typical DVSD present in ASD with the help of the model we designed in the previous section and examine the effect of training on its symptoms. Then, in “Experiments” Section, we present the results of the experiments. “Discussion” Section discusses the challenges and findings of this research and concludes the article.

## Related works

The visual system is a hierarchy of interconnected cortical areas. Previous studies on visual system modeling by deep learning techniques have often modeled and analyzed one of the visual streams. Moreover, the relationship between functions such as motion processing and perception of direction, heading, and speed in the dorsal stream has developed computational and experimental models^34–36^. Recently, significant progress has been made in modeling the ventral stream as a classifier. CNNs are state-of-the-art models of the ventral stream. In^21,22^, the authors state a strong correlation between a model’s performance in an object recognition task and its ability to predict cortical spiking data. Using this idea, they optimize the performance of the CNN model in the object recognition task. As a result, the model predicts neural activity in the ventral stream regions without the need for training with neural data.

Feedforward CNNs yield accurate models of the ventral stream function, but they are biologically different as they lack recurrent mechanisms. Local recurrence and long-range feedbacks explain dynamics in the primate visual system^37–39^. In^23,40^, authors study the impact of adding recurrences and feedback to CNNs on creating a more accurate and biologically plausible model of the ventral stream.

In^21–23^, authors use supervised methods and large amounts of labeled data to train their models. However, humans do not naturally possess access to such a large amount of labeled data for achieving visual abilities such as object recognition. In^41^, Zhuang et al. explain how humans learn these visual abilities in the first place using unsupervised methods. These methods are more adaptable to the biological functionality of the brain and achieve an accuracy that can compete with supervised methods. The color information was segregated from shape information in parallel streams of the CNN such as the primary visual cortex (V1) and secondary visual area (V2), and the color stream was related to the classification of animate images^42^.

Generative Adversarial Network (GAN), as an unsupervised method, has recently achieved high accuracy in generating realistic images^43^. GANs can simulate a population of neurons^44^. In^45^, authors combine a GAN with a Recurrent Neural Network (RNN) model. Then it processes electroencephalography (EEG) signals captured while subjects look at images. This method uses the EEG signal to condition a GAN to generate realistic images.

In the past, the dorsal stream modeling received less attention than the ventral stream. Of course, some previous works have modeled the dorsal visual stream. In particular, some studies such as^46,47^ have developed computational and experimental models of the dorsal stream. Then, with the help of these models, they investigated the relationship between functions such as motion processing and perception of direction, heading, and speed with the dorsal stream. In^48^, the model was proposed based on CNN models that show both a model ventral and dorsal pathway separately trained to do object and spatial recognition. In addition, models were proposed in^49^ that can accurately and simultaneously recognize and localize multiple objects in a scene. In^50^, the authors present a goal-driven model focused on the objectives of the dorsal stream. This model estimates self-motion parameters with the help of an Artificial Neural Network (ANN). Many of the previous works focus on modeling the localization abilities of the dorsal stream, as well as the object detection and classification functions of the ventral stream.

As explained in the previous section, patients with ASD often suffer from various DVSDs. In the context of ASD, the use of artificial intelligence techniques in previous studies has focused on the diagnosis of ASD. Some of these studies have identified people with ASD using physical characteristics such as facial attributes^33^. Another group of studies uses their behavioral and hereditary characteristics, such as a family history of ASD and eye-tracking, to identify people with ASD^30,31^. Some studies have also used functional magnetic resonance imaging (fMRI) of the brain to diagnose ASD^32^.

In this study, we present a model of the visual system using CNNs. Unlike previous models that only mimic one stream, our model consists of two visual streams. One of these streams mimics the object recognition task of the ventral stream. The other stream estimates the distance between objects as an example of the dorsal stream function. Therefore, we introduce a more biologically plausible model of the visual system. With the help of this model, we simulate the occurrence of a visual system disorder in ASD. We then examine the role of learning in improving the visual symptoms of this disorder. The differences between the previous works and VeDo-Net are outlined based on the visual pathway modeling characteristics in Table 1.

**Table 1:** Comparison of existing AI-based models for the visual system: tasks and features.

Articles	Features								
	CNN based	Classification	Location	Distance estimation	Multi-task	Dorsal pathway modeling	Ventral pathway modeling	Autism	Goal-driven
Yamins et al. (Hierarchical modular optimization (HMO) [2014])^21^	$\checkmark$	$\checkmark$					$\checkmark$		$\checkmark$
Nayebi et al. (Task-optimized ConvRNNs [2018])^23^	$\checkmark$	$\checkmark$					$\checkmark$		$\checkmark$
Sereno et al. (Attention in Dorsal Stream [2018])^36^						$\checkmark$		$\checkmark$	
Kietzmann et al. (Recurrence in Visual Cortex [2019])^37^	$\checkmark$	$\checkmark$					$\checkmark$		
Lindsay et al. (CNN for Visual Tasks [2021])^19^	$\checkmark$	$\checkmark$					$\checkmark$		
Zhuang et al. (Unsupervised neural network models [2021])^41^	$\checkmark$	$\checkmark$					$\checkmark$		
Han et al. (Modeling of Ventral & Dorsal Pathways [2023])^48^	$\checkmark$	$\checkmark$	$\checkmark$			$\checkmark$	$\checkmark$		$\checkmark$
Tamura et al. (Information segregation of CNN model [2024])^42^	$\checkmark$	$\checkmark$					$\checkmark$		
Han et al. (Modeling of Ventral & Dorsal Pathways [2024])^49^	$\checkmark$	$\checkmark$	$\checkmark$		$\checkmark$	$\checkmark$	$\checkmark$		$\checkmark$
The proposed VeDo-Net model	$\checkmark$	$\checkmark$	$\checkmark$	$\checkmark$	$\checkmark$	$\checkmark$	$\checkmark$	$\checkmark$	$\checkmark$

## Methods

In this section, we propose a model for the ability of the vision system to recognize objects and estimate the distance between them. We describe the details of the model in “VeDo-Net: the proposed model” Section. This model allows us to further investigate these visual tasks especially in certain impairments. Some neuro-diseases, such as ASD, can potentially damage the visual streams, which affects how visual information is processed. In this regard, in “Modeling the synaptic disruptions of dorsal visual stream” Section, we demonstrate how our suggested model can be used to reflect the impacts of ASD on the vision system.

### VeDo-Net: the proposed model

Vision is regularly the leading and crucial sensory modality in spatial cognition and object recognition due to its exceptional spatial resolution ^51^. The distinction between egocentric and allocentric frames of reference, one division in the study of spatial cognition, is inherently connected to the separation between categorical and coordinate spatial interactions^1^. Visual–spatial perception (VSP) is the capacity to correctly perceive an object’s physical location with respect to oneself and to recognize the physical relationship between other objects. VSP specifically explains how to evaluate an object’s size, shape, location, and motion^6–9^. The primary visual cortex processes visual stimuli in two parallel streams and generates visual perception^52,53^:

- The ventral visual stream or *what stream* (from the occipital cortex into the temporal cortex) underlies object recognition abilities. This stream supports color, texture, shape, and orientation processing^2,3^.
- The dorsal visual stream or *how/where stream* (from the occipital cortex into the parietal cortex) processes the spatial relationships between objects and motion. The dorsal stream processes visual–spatial attributes of the visual input^4,5^.

**Fig. 1:**
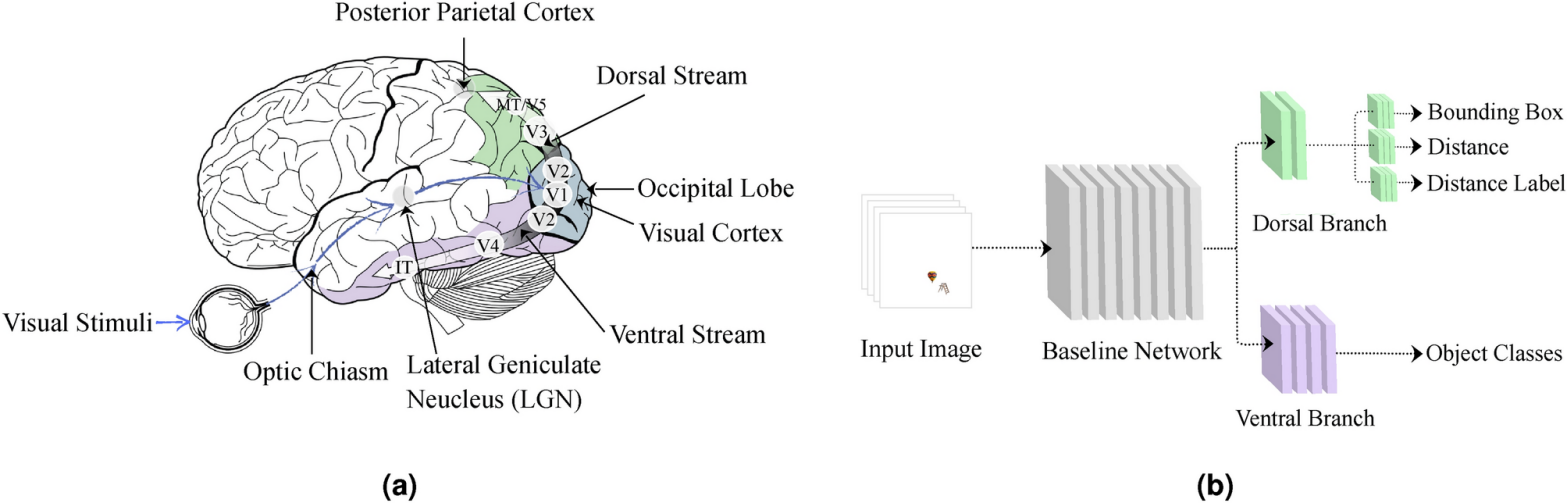
Schematic of the overall framework of the CNN-based visual system model architecture. (**a**) After the eye receives visual inputs, these inputs pass through the LGN to the visual cortex in the occipital lobe. The brain processes visual information along this way. After reaching the visual cortex, this information flows in two parallel streams. The ventral stream identifies the components of the input image. The dorsal stream creates an understanding of the spatial relationship between these components. (**b**) Dataset images are inputs to our model. Each part of the CNN-based model contains some layers. The baseline network processes the input image and extracts features from it. These features then flow in two parallel branches. The ventral branch identifies objects and generates their class as output. The dorsal branch outputs the location and distance between the objects. Figure created by the authors using draw.io software.

Figure 1a depicts the processing of visual stimuli in the visual system. The eyes receive visual information from the environment. This visual information enters the optic nerve after being processed in the retina. Then passing through the optic chiasm, the optic nerve sends this information to the thalamus’s lateral geniculate nucleus (LGN). The LGN transmits this information to the primary visual cortex or V1. After exiting V1, this information is processed in parallel in two streams. The ventral visual stream (V2 → V4 → inferior temporal (IT) cortex) is responsible for object recognition and discrimination. The dorsal stream (V2 → V3 → middle temporal area or MT/V5) processes spatial relationships and motion. Neurons in each visual cortex region can respond to different stimuli, but those stimuli are within a certain range. While the processes involved in these areas may seem simple, they can lead to complex visual perceptions when put together^54–56^.

This structure and function inspire us to model the visual system using CNNs. According to earlier research, CNN may be one of the most accurate computational models available for simulating brain activity^19,22,57^. We adopt a goal-driven approach in this modeling as the authors did in studies^21–23^ to achieve the highest accuracy in mimicking the tasks of the ventral and dorsal visual streams. The consistency of patterns of explicitly decodable information available to support behavioral activities (for example, object recognition, distance estimation, and so forth) serves as an effective metric of model resemblance to a system at the high-level task^22^. Typically, images forwarded into these networks are first normalized and divided into three channels (RGB) by capturing specific computations made by the retina. Following that, each stacked bundle of convolution layers is considered an approximate representation of a single visual area. Figure 1b demonstrates a schematic of the overall framework of the Ventral-Dorsal Network model’s (VeDo-Net model) architecture.

VeDo-Net model consists of a baseline network and two parallel branches like the visual system. Like the ventral stream, one of these branches recognizes objects(The terms branch and stream are used interchangeably to describe the proposed models’ architecture and to describe the anatomy of the visual system, respectively). Like the dorsal stream, the other branch processes spatial relationships between objects. This stream identifies the location of objects in the image. It also calculates the distance between them as an example of a spatial relationship. Table 2 shows the relationship between the different parts of our proposed model and the visual system.

The inputs to the human visual system are the scenes seen by the eyes. With regards to our model, the set of images used as input has imitated the visual input. Artificial neurons in our model play the role of biological neurons in the brain. As we know, brain neurons communicate with each other through synapses. In our model, weight vectors establish this connection between layers.

**Table 2:** Visual system versus the proposed VeDo-Net model.

	Visual system	VeDo-net model
Input	Visual Stimuli	Images
Unit	Biological Neurons	Artificial Neurons
Connection	Synapse	Weight Vector
Activation	Action Potential	Activation Function
Streams	Ventral and Dorsal stream	Two streams based on CNN
Output	Object Recognition, Estimation of Distance Between Objects, Location of Objects	Class of Object, Bounding Boxes, Distance

**Fig. 2:**
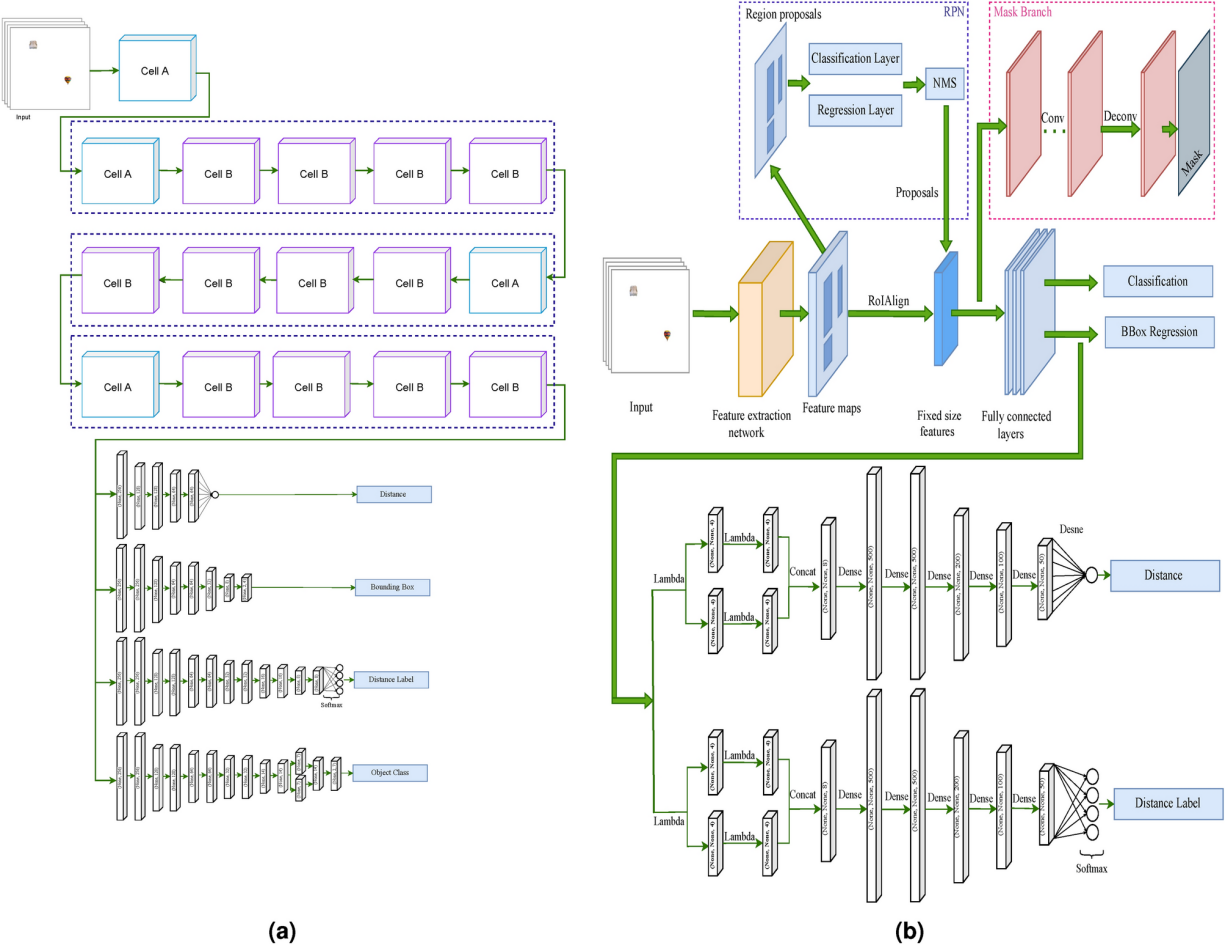
The architecture of the models. (**a**) The initial model uses NASNetMobile as the baseline network to extract features of the input image. NASNetMobile architecture’s Cell A is based on a reduction cell, and Cell B is based on a normal cell of^58^. These features are then processed by the four branches of CNN to produce appropriate outputs. (**b**) The improved model employs the Mask R-CNN network as the baseline to increase accuracy. In addition to feature extraction, this network produces outputs related to bounding boxes and object classes. We have added two more CNN branches to this model to calculate distance, and distance labels accordingly. The Lambda layers in both the distance and distance label branches work similarly. The first Lambda layer extracts the coordinates of the first and second detected objects in each image, as there are only two objects per image in our dataset. The other two Lambda layers normalize the bounding box coordinates for further processing or analysis, ensuring that the boxes are in a consistent coordinate system. Figure created by the authors using draw.io software.

Therefore, our main goal here is to build a model that has a structure similar to the visual system (Fig. 2a). The baseline of this model is a CNN, which serves as an all-purpose feature extractor. Two parallel branches separate after the baseline to process the extracted features in accordance with their respective tasks. The first branch includes one CNN branch that performs a classification task of the ventral stream^1^, i.e., to identify objects in the image. The second branch includes three CNN subbranches. One of these subbranches identifies the location of instances by estimating object bounding boxes. The other subbranch performs distance estimation as another task of the dorsal stream^1^. This subbranch uses regression to estimate the distance in pixels, and finally, the third subbranch performs a classification task to generate appropriate distance labels for each image.

In this paper, we constructed the feature extractor branch of the proposed model based on the model of NASNetMobile^58^ and two subbranches based on the model of hierarchical convolutional neural networks (HCNNs)^22^ to evaluate the performance of the proposed model. NASNetMobile architecture (neural architecture search neural network), was released by Google Brain in 2018, which is based on convolutional cells and serves as a useful model for classification tasks^23^. We loaded weights pre-trained on ImageNet for the feature extractor of the model. Then, we trained the subbranches of the model in a supervised manner. For training the model, we required a specific dataset. In “Dataset” Section, we introduce our dataset and explain the procedure we have carried out for generating it. Using this dataset, we determine the parameters of the model such that a minimal error occurs in each of the introduced outputs. We train the model with an initial learning rate of 0.003 and a batch size of 32 for 200 epochs. The size of each input of VeDo-Net model is 480 × 480.

Changing the architecture of the feature extractor of VeDo-Net model may lead to improvements to this state-of-the-art model. In this approach, we focus on the performance of VeDo-Net model to achieve a model that simulates the functions of the ventral and dorsal streams with high accuracy. To improve the initial model, we replace NASNetMobile with a baseline network that has more processing power. For this purpose, we present a visual system model that utilizes Mask R-CNN as the baseline network. We refer to this improved model as the VeDo-Net^+^ model, which is shown in Fig. 2b. Mask R-CNN is an extension of the Faster R-CNN object detection network^59^. Mask R-CNN can generate a segmentation mask for each object in the image in addition to the outputs of the Faster R-CNN, such as classifying and positioning objects^60^.

The classification of the instances in the image by Mask R-CNN mimics the task of the ventral stream. Finding the location of objects through generating the bounding boxes is also the task of the dorsal stream. In order to simulate the task of estimating the distance between samples as another task of the dorsal stream, we add two branches at the end of Mask R-CNN. One of these branches estimates the distance between objects in pixels by applying regression. With the help of a classification operation, the other branch produces a descriptive understanding of distance by assigning the appropriate distance label to the image.

### Modeling the synaptic disruptions of dorsal visual stream

Abnormalities of the visual system can lead to various outcomes, including disruptions in the brain’s visual processing, vision issues brought on by poor regulation of eye movement, and focus abnormalities. Most neuro-diseases, caused by genetic mutations or environmental factors, impair visual information processing by damage to the visual streams. Difficulties in recognizing objects, faces, shapes, and colors are typical symptoms of ventral stream damage. Damage to the ventral stream also impairs language perception, facial expressions, reading, and visual memory. Poor ability to handle complex visual scenes, reduced visual field, impaired visual guidance, perception of movement, and simultaneous perception are deficits in the dorsal stream damage^10,11^. Moreover, distance perception deficits and deficits in size constancy related to Dorsal Visual Stream Dysfunction (DVSD) can be present in conditions such as premature birth, cerebral palsy (CP), hydrocephalus, ASD Spectrum Disorder (ASD), Alzheimer’s disease (AD)^10,12,14^.

Children in particular experience vision impairments in several disorders, such as ASD. Malfunctions of the dorsal visual stream can affect how children interact with objects in their environment as well as how to understand the spatial relationships between them and with people and other objects^13^. ASD is a neuro-developmental disorder that manifests itself in the early years of a child’s growth. Children with ASD may have difficulty in sensory processing, movement awareness, and visual attention (such as contrast sensitivity, color perception, photosensitivity, and face recognition). The occurrence of ASD is due to abnormalities in the structure, activation, and connectivity of the brain areas compared to healthy individuals^61^. Patients with ASD may also exhibit disturbances in the functions related to spatial awareness and visuospatial and visual-motor processing, which indicate damage to the dorsal stream^62^. Some ASD patients tend to overestimate the distances with typical perceptual organization processes^63^. The connectivity and processing of the middle temporal visual area (MT/V5) is one of the fundamental drivers of individual differences along the ASD spectrum^64^. Motor functioning and other vision-related deficits in ASD are associated with DVSD^65,66^. Altered synaptic operations, including changing excitatory or inhibitory synaptic function, also play a substantial role in the development of ASD^67,68^. Studies show that methods such as training individuals with ASD through vision therapy help to enhance abnormalities across the visual system to some extent^69,70^.

DVSD in a child can result in a range of cognitive visual problems occurring every day, which is a major issue in children with neuro disabilities. Children with developmental coordination disorder (DCD) experience difficulties with motor imagery and distance estimation, which are also seen in children with CVI. Dorsal stream dysfunction is one of the most common ways children are affected by CVI^71,72^. Impaired visual–spatial perception, impaired visual guiding of movement, and simultaneous perception are some of the dorsal stream deficiencies seen in CVI an in general in DVSD.

Visual acuity is a measurement of the eye’s capacity to identify shapes and minute details of objects at a specific distance. The decreased visual acuity in a person with ASD leads to problems in social and repetitive behavior such as incorrect body posture or orientation, misunderstanding of the environment, poor eye contact, uncoordinated eye movement, and impaired reaching-grasping task^69,73^. In children with CVI, there is a relatively minor loss in visual acuity^71,72,74^. Atypical dorsal stream function is one of the most common symptoms of ASD. With the help of training and vision therapy, we can improve its behavioral signs in individuals with ASD or CVI^75,76^.

In this section, we model the incidence of synaptic dysfunction in the dorsal stream in ASD using the model presented in “VeDo-Net: the proposed model” Section. Then, with the help of this modeling, we examine the role of learning (i.e., by targeted training) in improving visual symptoms of this impairment in patients with ASD.

The Vedo-Net^+^ model can be similarly mapped to the visual system according to Table 2 which was presented earlier for the Vedo-Net model. The fully trained Vedo-Net^+^ model works the same way when the visual system is in a healthy state. The training process has determined the parameters of this model and the weight of the layers in such a way that the model can perform the dorsal and ventral stream tasks with minimal error.

In order to simulate synaptic dysfunction in the model, we change the values of the weight vectors between the layers of its dorsal branch. This change disrupts the communication of neurons in different layers of the network. As a result, it diminishes the performance of the dorsal and ventral branches. The situation is similar to when the visual system is in an unhealthy state.

The amount of change we make in weights determines the amount of damage done to the visual system model. The more weight vectors change, the further the model is from being ideal. As a result, the model needs more training to achieve its initial state. To simulate the early stages of ASD and CVI, we make fewer modifications to the weight vectors. We alter the weight vectors less to simulate the early stages of ASD and CVI. To simulate their advanced stages of them, we make more modifications to the weight vectors.

We demonstrate through simulation how well the suggested model retraining can imitate the training procedure used in vision therapy. We, therefore, retrain the model for 50 epochs in each case of modified network weights to ascertain if training can lessen dorsal impairment symptoms in accordance with the severity of diseases in our model.

## Experiments

In this section, we provide the results of our modeling scenarios explained in the previous section. For this investigation, we need a dataset consisting of images that include two distinct objects with a predetermined distance. In “Dataset” Section, we introduce the dataset we created in this regard to use in our study. Based on which, the results for the proposed models are then presented in “Results” Section.

### Dataset

**Fig. 3:**
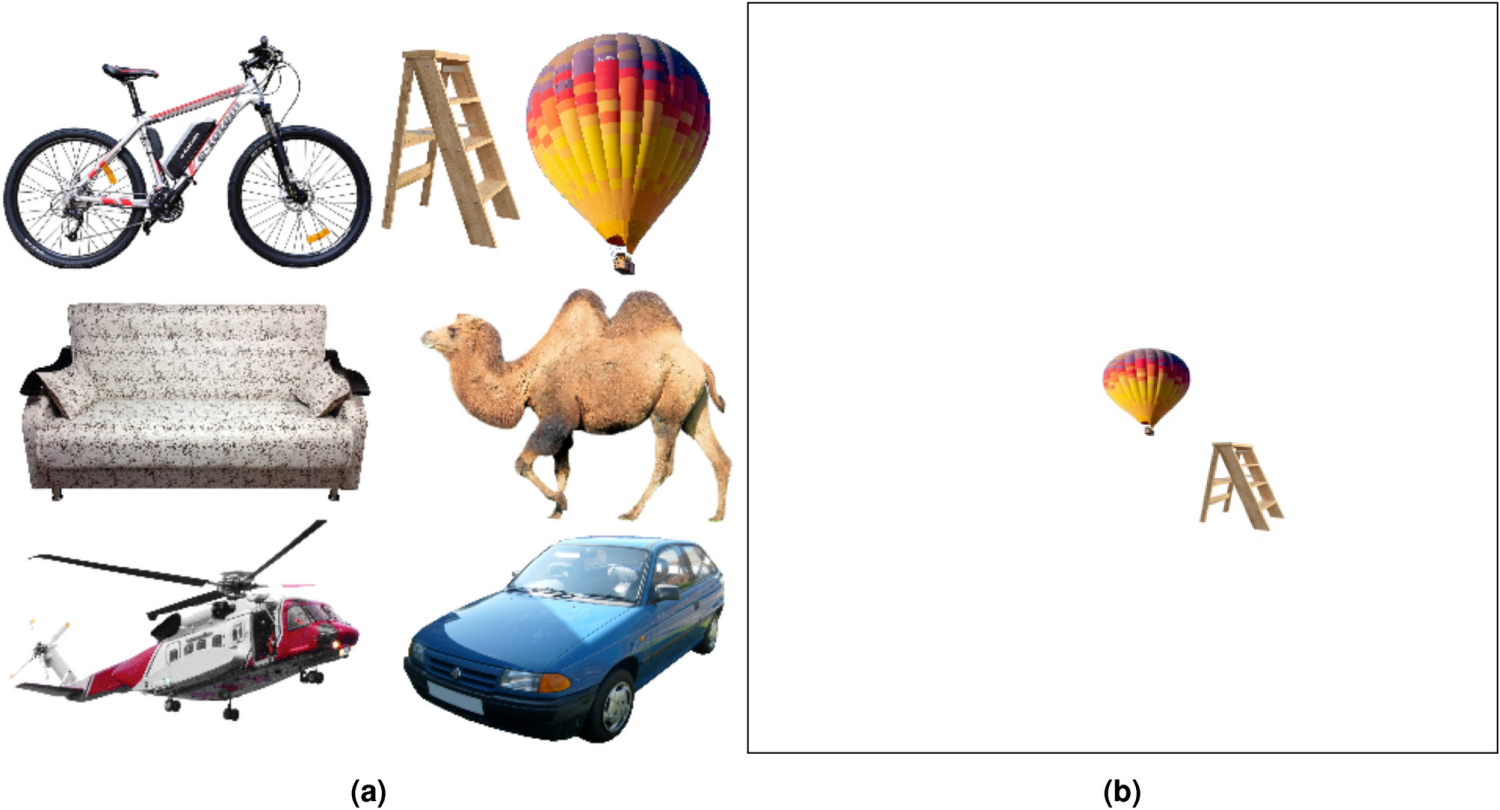
(**a**) The set of 7 objects in the dataset images. The camel figure under Creative Commons Zero License for Public Domain (https://www.needpix.com/photo/download/842129/), the car figure under the Creative Commons Attribution-Share Alike 3.0 Unported license (https://commons.wikimedia.org/wiki/), the balloon figure for Free use under the Pixabay Content License(https://pixabay.com/photos/), the bike figure under the Creative Commons CC0 1.0 Universal Public Domain Dedication license(https://picryl.com/media/), the ladder figure under Creative Commons Zero License for Public Domain (https://www.needpix.com/photo/), the sofa figure under Creative Commons Zero License for Public Domain (https://www.peakpx.com/441211/), and the helicopter figure under Free Personal and Business usage license (https://www.rawpixel.com/image/) are used. (**b**) One example of dataset images with two objects (the balloon figure for Free use under the Pixabay Content License(https://pixabay.com/photos/) and the ladder figure under Creative Commons Zero License for Public Domain (https://www.needpix.com/photo/)). The size of each image is 480 × 480 pixels and the size of each object is 60 × 60 pixels.

Our study proposes a model that can classify objects, determine the location of objects, and estimate distances between them. The characteristics of this model necessitate that the training data contains information regarding the aforementioned features. For this purpose, we have created a dataset for training and testing models (inspired by^77^).

Each image in this dataset contains two objects, which are randomly positioned on a white background of 480 by 480 pixels as shown in Fig. 3b. Each image has some annotations about the objects’ category, location, and distance saved in an XML file. Their location indicates a bounding box around the object.

We organize the dataset into 7 categories: balloons, bicycles, camels, cars, helicopters, ladders, and sofas. The bounding box around each object shows the coordinates of that object in the image. We consider the Euclidean distance between the centers of boxes of two objects as the distance between them. Although this distance is in pixels, we can train the models with real-world data if available.

On the other hand, Humans are able to qualitatively estimate how far or close objects are to one another when they look around. To address this concept, images also receive appropriate distance labels for different distances. If the distance between two objects in an image is between 0 and 120 pixels, we label the image as *Very Close*. If the distance between two objects in an image is between 120 and 240 pixels, we label the image as *Close*. If the distance between two objects in an image is between 240 and 360 pixels, we label the image as *Far*. If the distance between two objects in an image is more than 360 pixels, we label the image as *Too Far*. There are a total of 480 images in the dataset, with 120 images per class.

The dataset generation process can thus be summarized as follows:

- Randomly select two objects from a set of 7 objects as shown in Fig. 3a.
- Select two random points on the 480 × 480 white background.
- Place the selected objects at these two points.
- Calculate the coordinates of bounding boxes, the distance between their centers, and the distance label for each object.
- Save the generated image in .jpg format and the annotation information in .xml file.

**Table 3:** Visual system modeling results.

	VeDo-Net^+^ model
Object class accuracy	100%
Distance label accuracy	100%
MAE of distance (pixels)	0.54
IoU of bounding box	96.51%

### Results

We train and evaluate the performance of the proposed models use three metrics: the *accuracy* of label prediction, *Intersection of Union* (IoU), and *Mean Absolute Error* (MAE). We calculate the difference between the total distance and the predicted distance by MAE and compute the ratio of overlap between the total box and the predicted box by IoU. In other words, IoU measures the accuracy of object detection. We divide the dataset into training and testing sets with an 80/20 split, resulting in 384 images for training and 96 images for testing. We train our model using the dataset introduced in “Dataset” Section with an initial learning rate of 0.001 for 300 epochs.

The VeDo-Net model’s accuracy in classifying objects and distances is 76.04% and 97.91%, respectively. This model estimates the location of the boxes with an IoU of 32.85%. Also, the MAE of the model in estimating the distance is 10.19 pixels.

**Fig. 4:**
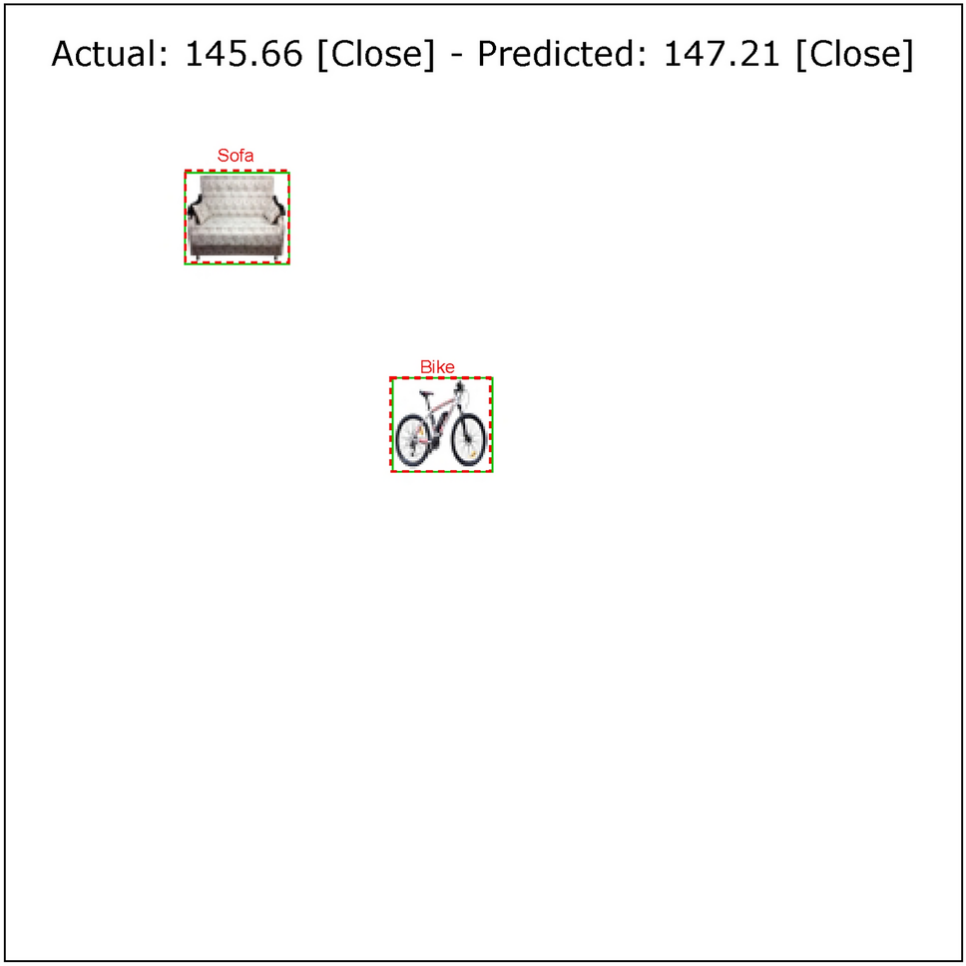
The output of VeDo-Net^+^ on the image with two objects(the sofa figure under Creative Commons Zero License for Public Domain (https://www.peakpx.com/441211/) and the bike figure under the Creative Commons CC0 1.0 Universal Public Domain Dedication license (https://picryl.com/media/)).

The results of the VeDo-Net^+^ model in a typical setting (after the training step), similar to the visual system in a healthy environment, are summarized in Table 3. The VeDo-Net^+^ model’s accuracy in classifying objects and distances is 100%. This model estimates the location of the boxes with an IoU of 94.56%. Also, the MAE of the model in estimating the distance is 0.88 pixels. The functionality of VeDo-Net^+^ is suitable for these tasks such as those shown in Fig. 4. As the results suggest, the VeDo-Net^+^ model outperforms the VeDo-Net model in all four visual system tasks. This model incorporates both dorsal and ventral branches, which are highly accurate in performing visual tasks.

**Table 4:** Modeling visual impairment Levels by adding random numbers to dorsal branch layers of the VeDo-Net^+^ Model.

	Initial values	Level 1		Level 2		Level 3	
		Impaired	Retrained	Impaired	Retrained	Impaired	Retrained
Distance label accuracy	100%	100%	100%	100%	100%	100%	100%
MAE of distance (pixels)	0.54	7.61	0.76	20.83	1.02	23.52	4.53
IoU of bounding box	96.51%	79.92%	95.41%	53.80%	93.20%	30.71%	92.16%

**Table 5:** Modeling visual impairment Levels by subtracting random numbers to dorsal branch layers of the VeDo-Net^+^ Model.

	Initial values	Level 1		Level 2		Level 3	
		Impaired	Retrained	Impaired	Retrained	Impaired	Retrained
Distance label accuracy	100%	100%	100%	70%	100%	45%	45.83%
MAE of distance (pixels)	0.54	10.76	1.1	119.81	67.38	181.67	104.89
IoU of bounding box	96.51%	58.61%	94.46%	6.63%	92.31%	0.44%	39.19%

**Table 6:** Modeling visual impairment Levels by adding and subtracting random numbers to dorsal branch layers of the VeDo-Net^+^ Model.

	Initial values	State 1		State 2	
		Impaired	Retrained	Impaired	Retrained
Distance label accuracy	100%	100%	100%	100%	100%
MAE of distance (pixels)	0.54	21.69	4.36	31.88	3.87
IoU of bounding box	96.51%	46.37%	93.84%	39.69%	94.28%

State 1 represents the scenario where positive changes in weights exceed negative changes, while State 2 represents the scenario where negative changes in weights are greater than positive changes.

In order to conduct further analysis, we apply the dorsal stream’s malfunction in our suggested model. To do this, we run the model while altering the weight of the last layers of the dorsal branches. After analyzing the model’s output, we retrain the model and assess whether the learning enhances the model’s functionality. We analyze the dorsal stream’s dysfunction on three levels (low, medium, and large). We alter the weights by adding or subtracting a random number to simulate each level of malfunction in the last layers of the VeDo-Net^+^ model. To simulate Level 1, we change the weights by adding/subtracting a small random number between 0 and 50% of their weights. Changing the weights by adding/subtracting a number between 50% and 75% of their weights indicates Level 2. We also simulate Level 3 by adding/subtracting the numbers larger than 75% of their weight to weight vectors. We change the VeDo-Net^+^ model’s weights in three levels to illustrate the evolution of dorsal dysfunction.

**Fig. 5:**
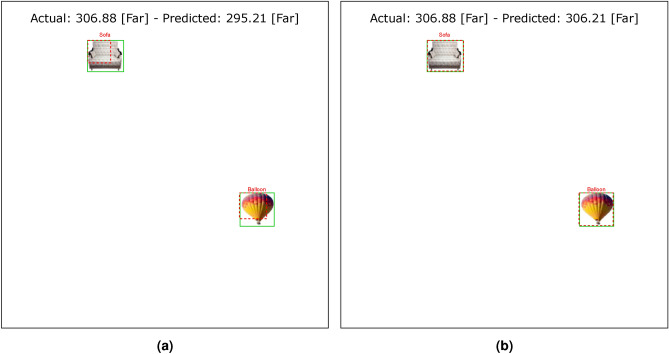
The output of Level 1 with negative changes in the weights of the dorsal branch’s last layers on the image with two objects. (**a**) This image shows examples of the output of an impaired model at Level 1. The model estimates the distance of objects less than the actual distance value. (**b**) This image depicts the results after retraining the model. As the picture shows, the effect of teaching in the early stages leads to great improvement. The sofa figure is under Creative Commons Zero License for Public Domain (https://www.peakpx.com/441211/) and the balloon figure is Free usage under the Pixabay Content License(https://pixabay.com/photos/).

**Fig. 6:**
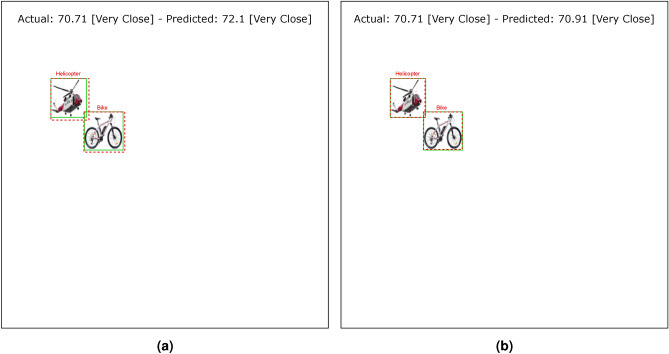
The output of Level 1 with positive changes in the weights of the dorsal branch’s last layers on the image with two objects. (**a**) This image shows examples of the output of an impaired model at Level 1. The model estimates the distance of objects more than the actual distance value. (**b**) This image depicts the results after retraining the model. As the picture shows, the effect of teaching in the early stages leads to great improvement. The helicopter figure is under Free Personal and Business usage license (https://www.rawpixel.com/image/9196648/) and the bike figure is under the Creative Commons CC0 1.0 Universal Public Domain Dedication license(https://picryl.com/media/).

**Fig. 7:**
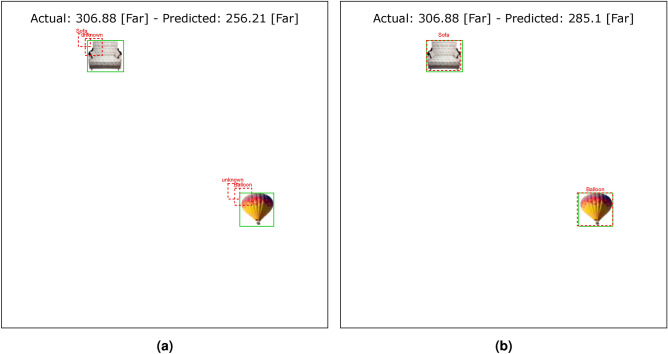
The output of Level 2 with negative changes in the weights of the dorsal branch’s last layers on the image with two objects. (**a**) This image shows examples of the output of an impaired model at Level 2. The model estimates the distance of objects less than the actual distance value. (**b**) This image depicts the results after retraining the model. The sofa figure is under Creative Commons Zero License for Public Domain (https://www.peakpx.com/441211/) and the balloon figure is Free usage under the Pixabay Content License(https://pixabay.com/photos/).

**Fig. 8:**
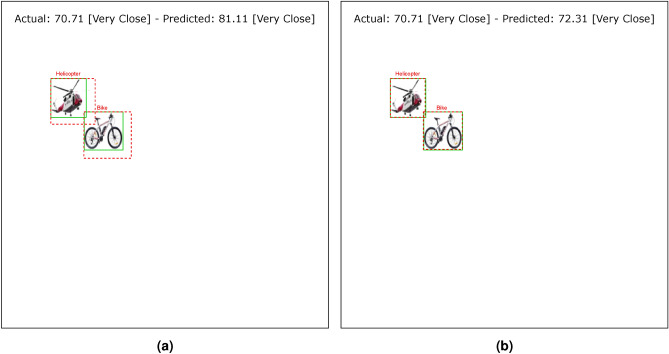
The output of Level 2 with positive changes in the weights of the dorsal branch’s last layers on the image with two objects. (**a**) This image shows examples of the output of an impaired model at Level 2. The model estimates the distance of objects more than the actual distance value. (**b**) This image depicts the results after retraining the model. The helicopter figure is under Free Personal and Business usage license (https://www.rawpixel.com/image/9196648/) and the bike figure is under the Creative Commons CC0 1.0 Universal Public Domain Dedication license(https://picryl.com/media/).

**Fig. 9:**
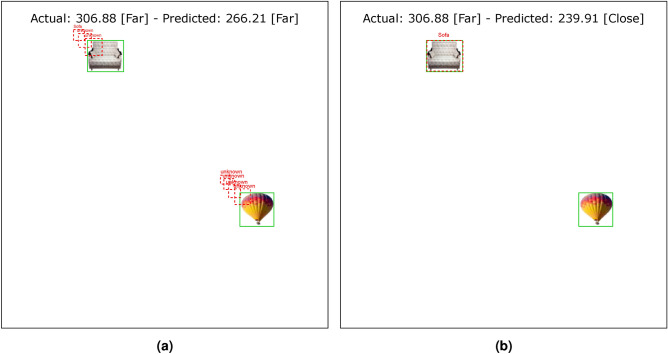
The output of Level 3 with negative changes in the weights of the dorsal branch’s last layers on the image with two objects. (**a**) This image shows examples of the output of an impaired model at Level 3. The model estimates the distance of objects less than the actual distance value. (**b**) This image depicts the results after retraining the model. The sofa figure is under Creative Commons Zero License for Public Domain (https://www.peakpx.com/441211/) and the balloon figure is Free usage under the Pixabay Content License(https://pixabay.com/photos/).

**Fig. 10:**
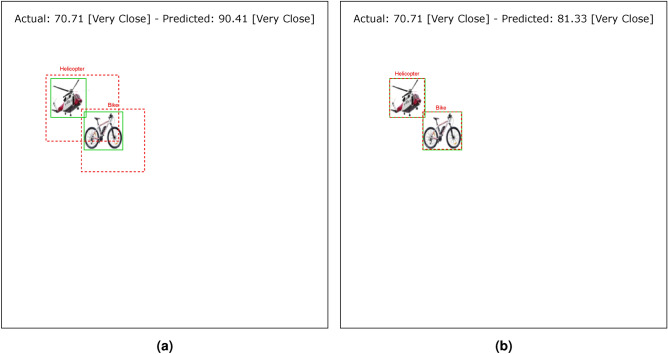
The output of Level 3 with positive changes in the weights of the dorsal branch’s last layers on the image with two objects. (**a**) This image shows examples of the output of an impaired model at Level 3. The model estimates the distance of objects more than the actual distance value. (**b**) This image depicts the results after retraining the model. The helicopter figure is under Free Personal and Business usage license (https://www.rawpixel.com/image/9196648/) and the bike figure is under the Creative Commons CC0 1.0 Universal Public Domain Dedication license(https://picryl.com/media/).

Tables 4 and 5 show the results of the simulation of dorsal branch impairment in DVSD. In the simulation of Level 1, after retraining the model, we achieve a very close accuracy to the initial accuracy of the model (Figs. 5 and 6). In this case, the training compensates for the disturbances caused by the weight changes (representing the impairment) in the outputs of the model dorsal branch. In modeling Level 2, the model accuracy increases after changing weights due to training (Figs. 7 and 8). In this case, since there are more changes in the weight vectors, the compensatory effect of the training results in less improvement than in the first case. In modeling level 3, retraining improves the model accuracy, but this improvement is so slow that it has no significant effect on the model outputs as it can be seen in Figs. 9 and  10. Based on the results, it appears that the negative impact of subtracting random numbers is more than that of adding random numbers. Weakening the connection between layers complicates the VeDo-Net^+^ model’s performance. So, the VeDo-Net^+^ Model’s accuracy is less than 100 in Levels 2 and 3 (Table 5). In other words, the effect of the existence or nonexistence of neurotransmitters is not symmetric on synaptic functionality. Almost neuro-diseases occur based on a lack of some neurotransmitters such as seizure, Alzheimer’s disease, Parkinson’s disease, depression, schizophrenia, and so on^78,79^.

**Fig. 11:**
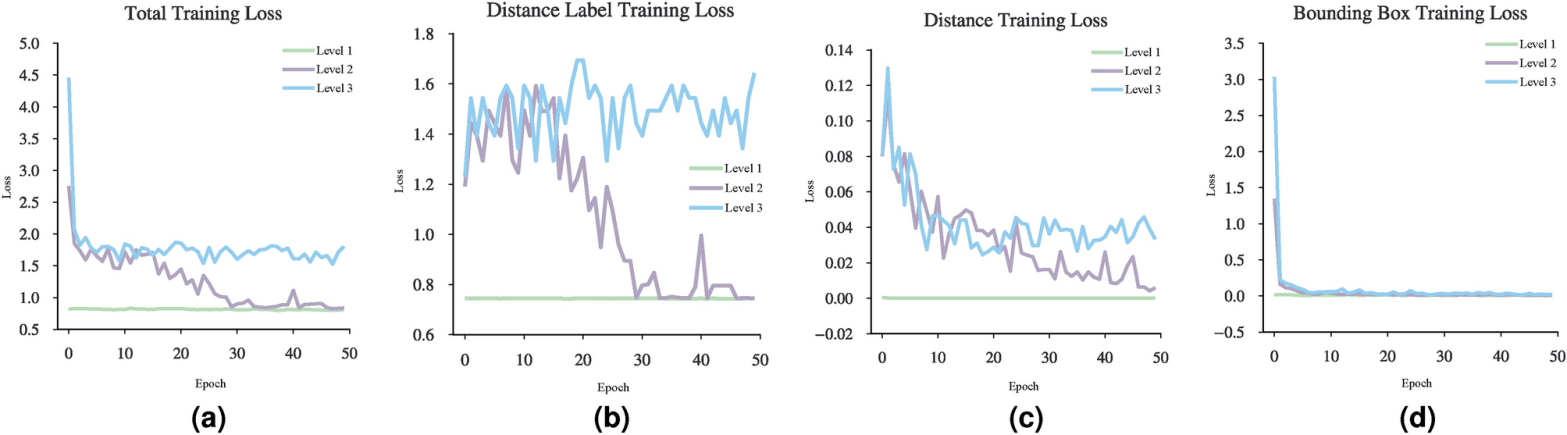
Loss function diagram in dorsal branch impairment simulation during model retraining. The model estimates the distance of objects as less than the actual distance value. The more severe the dorsal branch impairment, the harder the retraining effect.

**Fig. 12:**
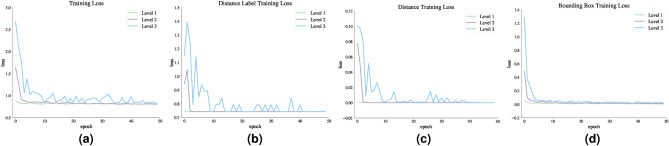
The loss function diagram in dorsal branch impairment simulation during model retraining. The model estimates the distance of objects as more than the actual distance value. The more severe the dorsal branch impairment, the harder the retraining effect.

Figures 11 and 12 show the loss function diagram during the retraining model. The amount of loss in all outputs related to the dorsal branch decreases during retraining. This supports the idea that targeted training by visual therapy improves dorsal branch impairment. In addition, for all outputs related to the dorsal stream, the loss function is greater after retraining with increasing dorsal branch impairment severity. This confirms that the effect of training and visual therapy in the severe stages of the impairment is less than in the early stages.

These results are consistent with the fact that in the mild stages of dorsal damage, training can help improve the visual symptoms. The more severe the disease, the less effective this training will be, as people in the advanced stages of dorsal damage (such as ASD) will need very serious support in their daily activities. Figs. 5, 6, 7, 8, 9 and  10 show some examples of the simulation results for the effects of dorsal impairment.

**Fig. 13:**
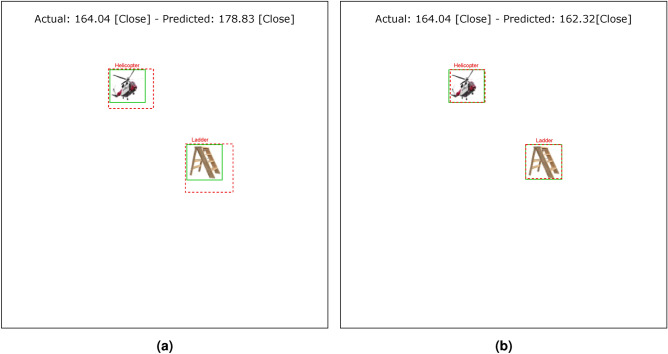
The result of the damaged model on the image with two objects. With the effects of both positive and negative changes, the weights of the last layer of the dorsal branch have been altered. Positive changes have a greater impact than negative ones in the weights. (**a**) Examples of a model’s output are shown in this image. The model estimates the distance of objects as more than the actual distance value. (**b**) The outcomes of the model’s retraining are shown in this image. The helicopter figure is under Free Personal and Business usage license (https://www.rawpixel.com/image/) and the ladder figure is under Creative Commons Zero License for Public Domain (https://www.needpix.com/photo/).

**Fig. 14:**
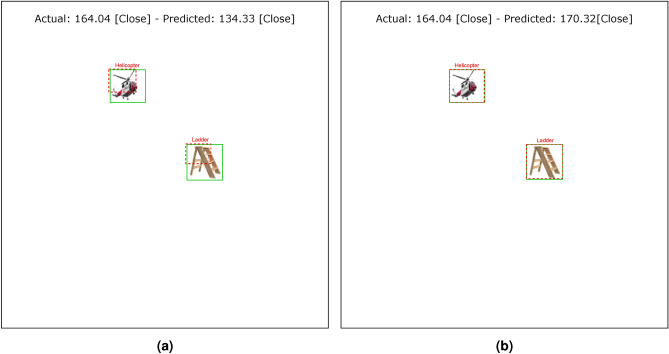
The result of the damaged model on the image with two objects. With the effects of both positive and negative changes, the weights of the last layer of the dorsal branch have been altered. Negative changes have a greater impact than positive ones in the weights. (**a**) Examples of a model’s output are shown in this image. The model estimates the distance of objects as less than the actual distance value. (**b**) The outcomes of the model’s retraining are shown in this image. The helicopter figure is under Free Personal and Business usage license (https://www.rawpixel.com/image/) and the ladder figure is under Creative Commons Zero License for Public Domain (https://www.needpix.com/photo/).

**Fig. 15:**
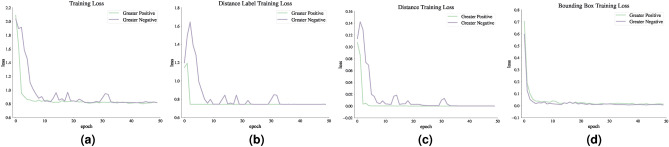
Loss function diagram in dorsal branch impairment simulation during model retraining. In this case, the weights of the last layer of the dorsal branch have been disturbed with ith the consequences of both positive and negative changes.

We ultimately model and analyze the combined effects of positive and negative changes in the weights of the last dorsal layers. The outputs of the model with the consequences of both positive and negative changes in the last dorsal layers are shown in Figs. 13 and 14. The model could overestimate or underestimate the distance between objects depending on how much the positive or negative changes have affected the model’s weights. Table 6 shows the results of the simulation of retraining of dorsal branch impairment in DVSD. The training in these situations makes up for the perturbations brought on by the relatively slight weight value changes in the model’s dorsal branch. Fig. 15 shows the loss function diagram during the retraining model. Retraining the network with a number epochs set beforehand, such that it returns to the initial performance level is not always feasible due to the variation of the levels of impairments. For some certain levels, it is possible that the impaired network would need to adjust its hyperparameters in addition to retraining to reach its initial performance level. This can be seen quite similar to Retraining exercises the duration of which vary depending on the person’s autism severity level^80^.

## Discussion

The discovery that artificial neural networks may recapitulate the representation of visual information along visual streams is one of the main reasons for the recent interest in these systems among computational neuroscientists. The ability to anticipate the activity of genuine neurons with more precision than earlier techniques is one of the benefits of these technologies.

Here, we explored the neurobiology of the ventral and dorsal streams of the vision system from a modeling perspective. We have demonstrated the connections of streams of the visual system through deep learning techniques and that CNN-based models can serve as a means to model and examine the system’s performance on a visual task. Each layer of the proposed model carries out straightforward tasks that are implementable in biological circuitry, like convolution, pooling, and normalization. We used backpropagation to train our proposed model to detect objects, estimate distances, and classify distance labels. As a result, the network autonomously learned neuronal tuning functions. To evaluate the performance of the human visual system and the VeDo-Net model, we compared the output of the VeDo-Net model and the human vision system in some different situations. Unlike previous studies, we modeled the two streams of the visual system simultaneously.

Our results indicate that our model matched the dynamics of neural activity in the ventral and dorsal streams of the visual system more satisfactory than vision networks^21–23,41^. Although we did not explicitly design these structures to resemble cortical anatomy, the presence of feed-forward connections between discrete neuronal populations is consistent with the known anatomy^21,22^. Our findings suggest that the functionality of two streams within the brain may be adapted for different behaviors. This model incorporates both dorsal and ventral branches, which are highly accurate in performing visual tasks.

Optimizing the proposed VeDo-Net model^60^ (based on faster RCNN^59^) for more sophisticated feed-forward CNNs could improve these state-of-the-art baselines. Thus we extended the core of VeDo-Net model with a Mask R-CNN model: given an appropriate architecture class, task-driven, Faster R-CNNs provide the best normative models of encoding dynamics of two visual system streams.

We explored, by further tests, how dorsal visual stream dysfunction in ASD could impair the functionality of dorsal stream. Our results were consistent with the fact that in the mild stages of ASD, targeted training via visual therapy can help improve the visual symptoms of ASD. Nonetheless, the more severe the disease, the less effective this training will be, as people in the advanced stages of ASD will need very serious support in their daily activities^81^.

## Data Availability

The code and datasets generated during the current study are available in the “VSMObjects Dataset” repository, [https://github.com/VeDo-Net/VeDo-Net].
